# Metabolic flexibility and reverse remodelling of the failing human heart

**DOI:** 10.1093/eurheartj/ehaf033

**Published:** 2025-02-25

**Authors:** Peregrine G Green, William D Watson, Benjamin M Bussmann, Giovanni Luigi De Maria, Stefan Neubauer, Andrew J M Lewis, Oliver J Rider, Neil Herring

**Affiliations:** Department for Physiology, Anatomy and Genetics, University of Oxford, Oxford OX1 3PT, UK; Oxford Centre for Magnetic Resonance Research, University of Oxford, Oxford, UK; Oxford Centre for Magnetic Resonance Research, University of Oxford, Oxford, UK; Department for Physiology, Anatomy and Genetics, University of Oxford, Oxford OX1 3PT, UK; Oxford Centre for Magnetic Resonance Research, University of Oxford, Oxford, UK; Department of Cardiology, John Radcliffe Hospital, Oxford University Hospitals NHS Foundation Trust, Oxford, UK; Oxford Centre for Magnetic Resonance Research, University of Oxford, Oxford, UK; Oxford Centre for Magnetic Resonance Research, University of Oxford, Oxford, UK; Oxford Centre for Magnetic Resonance Research, University of Oxford, Oxford, UK; Department for Physiology, Anatomy and Genetics, University of Oxford, Oxford OX1 3PT, UK; Department of Cardiology, John Radcliffe Hospital, Oxford University Hospitals NHS Foundation Trust, Oxford, UK

**Keywords:** Non-ischaemic cardiomyopathy, Metabolism, Reverse remodelling, Resynchronization

## Abstract

**Background and Aims:**

Cardiac resynchronization therapy (CRT) produces long-term reverse remodelling which requires greater adenosine triphosphate delivery to the contractile machinery. Whilst the heart retains some metabolic flexibility in non-ischaemic cardiomyopathy, whether this correlates with reverse remodelling is unknown. This study investigated whether CRT acutely changes cardiac substrate uptake, and whether this translates to favourable reverse remodelling.

**Methods:**

The effect of CRT on cardiac substrate uptake was assessed via direct coronary flow and arteriovenous measurements, with metabolomic/lipidomic analysis on infusions of insulin/glucose and intralipid. Cardiac function was assessed with left ventricular pressure–volume loops during implantation, and cardiac magnetic resonance before and 6 months following CRT, with and without biventricular pacing.

**Results:**

Regardless of substrate infusion, CRT acutely improved stroke work without increasing O_2_ uptake on both insulin/glucose (by 34%, *P* = .05) and intralipid (by 36%, *P* = .03). This was followed by increased fatty acid (FA) uptake on insulin/glucose (*R* = 0.89, *P* = .03) and increased β-hydroxybutyrate uptake (*R* = 0.81, *P* = .05) during intralipid infusion. After 6 months, there was a 48% (*P* < .001) reduction in left ventricular end diastolic volume, beyond that achievable by acutely shortening or lengthening QRS duration. Reverse remodelling significantly correlated with increased FA uptake with CRT on insulin/glucose (*R* = 0.71, *P* = .05) driven by long and medium chain uptake, and increased ketone uptake with CRT on intralipid (*R* = 0.79, *P* = .05).

**Conclusions:**

CRT acutely alters the metabolic phenotype of non-ischaemic cardiomyopathy towards a more physiological picture of FA uptake which correlates with reverse remodelling. Retained metabolic flexibility may therefore be critical for subsequent reverse remodelling.

## Structured Graphical Abstract

**structured graphical abstract:**
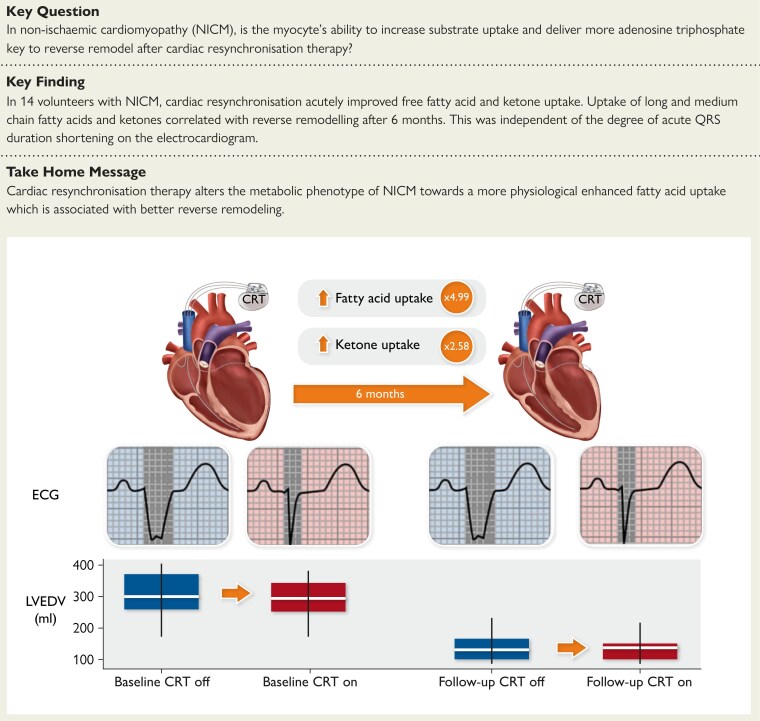
Cardiac resynchronization therapy acutely shortens the QRS duration on the electrocardiogram (blue to red), which leads to an increase in fatty acid and ketone uptake as shown above. Whilst this does not acutely change left ventricular end diastolic volume (LVEDV), after 6 months significant reverse remodelling of LVEDV occurs as shown in the lower graph, which correlates with the degree of fatty acid and ketone uptake but is not reversed by acutely turning CRT off to broaden QRS duration on the electrocardiogram (red to blue above).

**See the editorial comment for this article ‘Fatty acids in heart failure patients: friend or foe?’, by H. Wiggers, https://doi.org/10.1093/eurheartj/ehaf247.**

Translational perspective

- In non-ischaemic cardiomyopathy, cardiac resynchronization therapy (CRT) acutely improves cardiac haemodynamic performance, and subsequently increases the uptake of fatty acids and ketones which is associated with the degree of left ventricular reverse remodelling over 6 months.
- Whilst manipulating cardiac substrate usage can change cardiac function, this is the first study to show that improving cardiac function precipitates changes in cardiac substrate use, which may serve as a predictor of response to CRT. Therapies targeting pathways to increase fatty acid and ketone metabolism may form the basis of novel therapies to promote reverse remodelling in heart failure and improve CRT response.

## Introduction

Altered energy metabolism is a hallmark of heart failure (HF), with the heart likened to an ‘engine out of fuel’.^1^ There is reduced oxidative capacity in the mitochondria,^2^ and profound changes to the creatinine kinase system resulting in up to 70% reduced adenosine triphosphate (ATP) delivery to myofibrils.^3^ This is associated with an absolute reduction in both fatty acid (FA)^4^ and glucose^5^ uptake and oxidation, resulting from reduced expression of key regulatory genes in FA and glucose metabolism.^6,7^ Overall, due to a greater decrease in FA oxidation, a relative substrate switch towards glucose metabolism is observed.^7^ Historically, this switch towards glucose metabolism was considered a compensatory attempt to increase cardiac efficiency and ATP production from glycolysis in the presence of reduced oxidative capacity.^4^ Observations that the failing heart is unable to augment glucose metabolism in response to stress^8,9^ led to the concept of metabolic inflexibility contributing to the pathogenesis of HF. However, recent data in patients with non-ischaemic cardiomyopathy (NICM) demonstrate that the heart retains some metabolic flexibility in response to changes in arterial substrate supply and workload.^10^ In addition, increased FA but not glucose metabolism was associated with improved myocardial function while still maintaining sufficient oxygenation.^10^

In some circumstances, left ventricular (LV) systolic function can be restored if there is surviving myocardium and the underlying cause of the HF can be successfully treated. This process, known as ‘reverse remodelling’, is unpredictable and poorly understood. Cardiac resynchronization therapy (CRT) is a treatment for HF with a broad left bundle branch block (LBBB), where LBBB causes dys-synchronous contraction between the septum and lateral wall of the LV, reducing systolic function. CRT acutely improves cardiac haemodynamics and oxygen efficiency,^11^ although there appears to be further improvement in LV systolic function with reverse remodelling occurring over the first 6 months, leading to an improvement in symptoms and mortality in randomized controlled clinical trials.^12–14^ However, around 1 in 3 patients do not obtain an improvement in systolic function or symptoms and identifying those most likely to respond remains a challenge. Those with a longer QRS duration (QRSd), true LBBB, NICM, and fewer comorbidities appear to benefit most.^15^

On a cellular level, CRT has been shown to improve cell survival while increasing potassium currents, myocyte contractility, and β-adrenergic receptor responsiveness.^16^ However, the mechanisms through which CRT leads to long-term structural cardiac remodelling remain unclear. Some evidence suggests homogenization of cardiac work,^17^ changes in mitochondrial protein expression to promote mitochondrial efficiency and oxidative phosphorylation^18^ and changes in gene expression regulating contractile function.^19^ In the long term, CRT can change the circulating metabolome,^20^ although whether this is cause or effect is not clear. Ultimately, an improvement in contractile function necessitates greater ATP delivery to the contractile machinery. Therefore, it is plausible that the degree of metabolic flexibility retained by the failing heart may be key to its ability to reverse remodel.

This study therefore aimed to accurately characterize the degree of left ventricular reverse remodelling in response to 6 months of CRT compared with acute QRSd shortening, using paired cardiac magnetic resonance imaging (CMR) measurements. It then addresses the hypothesis that CRT can acutely change substrate uptake away from the metabolic phenotype of HF, which ultimately translates to favourable reverse remodelling. This is achieved through invasive measures of cardiac substrate, metabolomic and lipidomic uptake in patients with NICM undergoing CRT implantation, in relation to acute contractile performance measured with an LV pressure-volume catheter and chronic structural reverse remodelling using CMR.

## Methods

### Study population

**Figure 1:**
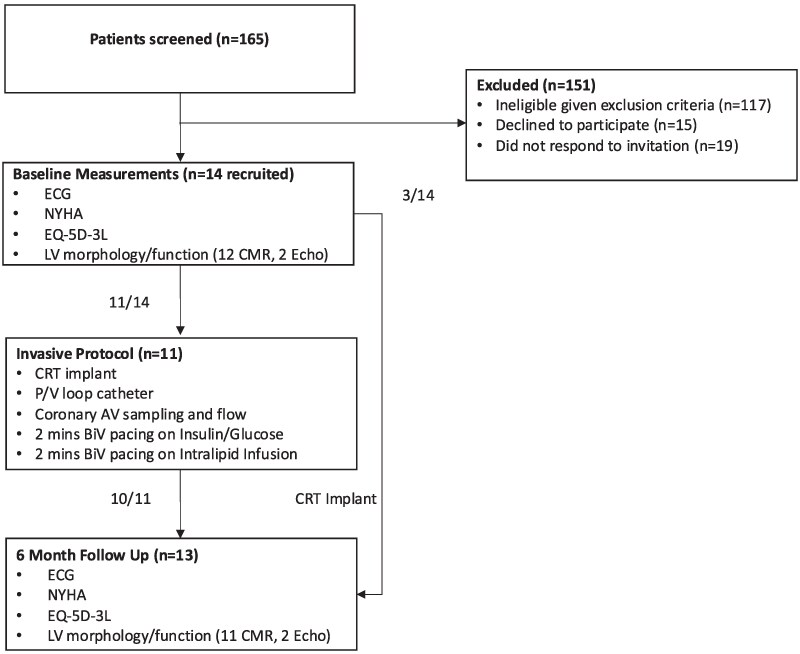
CONSORT diagram illustrating the overall study protocol. AV, arteriovenous; BiV, biventricular; CMR, cardiac magnetic resonance imaging; CRT, cardiac resynchronization therapy; ECG, electrocardiogram; Echo, transthoracic echocardiography; EQ-5D-3L, EuroQol 5-Dimension 3-Level score; LV, left ventricle; MVO_2_, minute volume of oxygen; NYHA, New York heart association class; P/V, pressure–volume

Fourteen volunteers with NICM scheduled to have clinically indicated CRT insertion according to European Society of Cardiology guidelines^21^ were recruited as described in the CONSORT diagram in *Figure 1*. Exclusion criteria were ischaemia as the primary cause of HF (infarction in one or more segments on CMR or not greater than mild coronary artery disease on coronary angiography, as defined by 50% luminal stenosis), egg or soy allergy, disturbances of normal fat metabolism (e.g. hyperlipidaemia, lipoid nephrosis, or pancreatitis), liver disease (e.g. imaging evidence of cirrhosis or liver function tests outside the normal range), recent fracture of pelvis or long bones, anaemia (defined as haemoglobin below the normal range), blood coagulation disorders (not including the use of oral anticoagulants), or metallic foreign bodies preventing imaging.

### Study protocol

Prior to CRT implantation, participants had baseline ECG, New York Heart Association (NYHA) class and EuroQoL 5-Dimension 3-Level (EQ-5D-3L) score recorded and a baseline cardiac CMR was performed (2 participants did not undergo baseline CMR and as such echocardiogram measurements were used in these participants). CMR was performed 2 weeks prior to CRT implantation in all except one patient where implant was delayed by 3 months due to the impact of the COVID-19 pandemic. During CRT implantation, 11 participants underwent an invasive protocol with PV loop and coronary flow measurements with paired coronary arterio-venous blood sampling. Participants were followed up 6 months after CRT implantation, with repeat CMR and assessment of ECG, NYHA class, and EQ-5D-3L score. Follow-up assessments of LV function with CMR were performed with both AOO pacing (‘Intrinsic’) and DOO with BiV pacing (in a randomized order), with asynchronous pacing set 10 bpm above the participant’s intrinsic heart rate to prevent competitive pacing. A flow diagram illustrating the overall study protocol is shown in *Figure 1*.

### Cardiac magnetic resonance imaging

Prior to CRT implantation, participants underwent CMR imaging performed at 3T (Tim Trio; Siemens Healthineers) with a short stack of cine images obtained using a 24-channel spine coil, and 6-channel body array (both Siemens), and steady-state free precession sequences, as described previously.^10^ Images were analysed with manually drawn LV endocardial and epicardial contours in cvi42 (Circle Cardiovascular Imaging).

### Invasive protocol during cardiac resynchronization therapy device implantation

**Figure 2:**
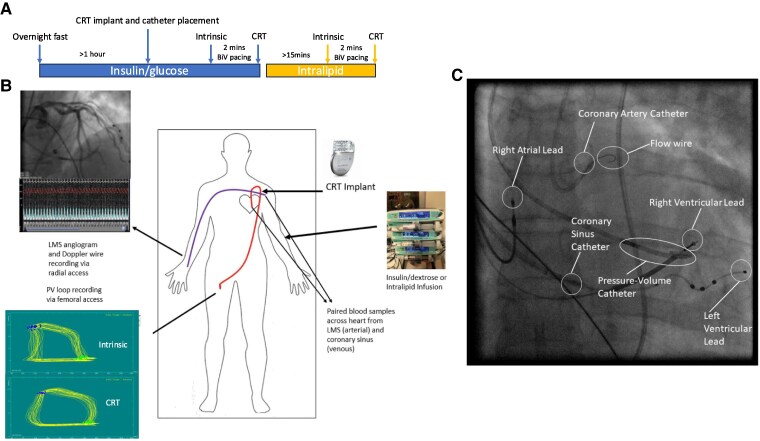
Schematic diagram of the invasive protocol. (*A*) Timeline of the invasive protocol after an overnight fast. Patients were stabilized on an insulin/glucose infusion for at least 1 h prior to cardiac resynchronization therapy device implantation and positioning of catheters. Pressure–volume loop measurements and coronary arteriovenous sampling was then carried out during intrinsic arteriovenous node conduction, and after 2 min of biventricular pacing. The insulin/glucose infusion was then stopped and an intralipid infusion started for at least 15 min, after which measurements were repeated. (*B*) Diagram showing the positioning of catheters and guidewires and measurements obtained during the invasive protocol during intrinsic arteriovenous conduction or cardiac resynchronization therapy. (*C*) fluoroscopic image illustrating the positioning of catheters, guidewires and pacing leads in a participant undergoing the invasive protocol

All measurements were taken during scheduled CRT implantation (Boston Scientific) after an overnight fast as shown in *Figure 2A*. Before the procedure, all patients were established on a euglycemic hyperinsulinaemic clamp with an insulin + glucose (I + G) infusion where the rate of insulin-infusion was adjusted during the glucose infusion to maintain plasma glucose ±1 mmol/L of baseline using an internally developed algorithm as described previously.^10^ After the implantation of the leads, the CS guide catheter was pulled back into the CS os for blood sampling, and the leads attached to the generator to enable programmable pacing. Radial arterial access was then established, and a 6 Fr guide catheter passed to the left main coronary artery to perform a coronary angiogram and position a flow wire (Volcano; Philips Healthcare) into the proximal segment of the left main coronary artery to measure coronary arterial flow velocity (cm/s). Coronary flow (mL/s) was then calculated as π×coronary radius^2^ (taken from fluoroscopy)×coronary arterial flow velocity. Eight Fr femoral arterial access was obtained and a conductance catheter (Inca; CD Leycom) was passed across the aortic valve to the apex of the LV, and intra-arterial heparin given to keep the ACT ≥250 s. 60 s of pressure volume loop measurements from the impedance catheter and coronary flow measurements from the flow wire were taken, followed by simultaneous paired coronary arterial (from the coronary artery catheter) and venous blood sampling (from the coronary sinus). This was performed during intrinsic rhythm and then following 2 min of biventricular pacing optimized using the Oxford method^22^ at intrinsic heart rate (atrial sensed). Previous studies have shown that changes in coronary flow and cardiac oxygen consumption in response to acute mechanical changes stabilize within 60–90 s.^23^ After these measurements were performed, the I + G infusion was stopped, and an infusion of 20% intralipid infusion started at 60 mL/hr. At least fifteen minutes after the beginning of the intralipid infusion, the above sampling protocol was repeated during intrinsic rhythm, and biventricular pacing as shown in *Figure 2A*. At the end of the protocol, a repeat coronary angiogram was performed to demonstrate no complications due to positioning of the flow wire, and catheters were removed and haemostasis obtained. Blood samples were assayed for oxygen saturation, partial pressure of oxygen, and haemoglobin using a blood gas analyzer (Seca). Glucose, non-esterified free fatty acids (NEFA), β-hydroxybutyrate (BOHB), insulin, and lactate levels were all assessed in the clinical-grade Oxford University Hospitals Clinical Laboratories. Metabolomic and lipidomic analysis was performed by a commercial provider (Metabolon, Durham NC, USA) and analysed as described previously.^10^

Myocardial oxygen usage (MVO_2_) was determined from arteriovenous content difference (0.0225×PO_2_ KPa + 1.4×[Hgb] × [%O_2_ sat])×flow rate [mL/s]) and myocardial carbon dioxide production (MVCO_2_) was calculated from the McHardy equation (11.02×[(PvCO_2_)0.396—(PaCO_2_)0.396]—(15–Hb)×(PvCO_2_–PaCO_2_)—(95–SaO_2_)×0.0064). Respiratory quotient (RQ) was calculated as cardiac VCO_2_/VO_2_. Absolute myocardial substrate uptake was determined from arteriovenous difference (glucose/lactate/ketone/NEFA)×coronary flow rate. Myocardial oxygen efficiency was defined as the ratio of cardiac work to MVO_2_ (L.mmHg/mL).

### Six month follow-up cardiac magnetic resonance imaging

Post-CRT implantation follow-up scans were performed in a Magnetom Avanto^TM^ 1.5 Tesla scanner (Siemens, Erlangen, Germany). All CRT devices were interrogated and programmed using a Zoom Latitude^TM^ programming system (Boston Scientific, Massachusetts, USA). Devices were set into an MRI safe mode as per the manufacturer’s instructions, with asynchronous pacing set 10 bpm above the participant’s intrinsic heart rate to prevent competitive pacing. ECG and pulse oximetry monitoring was used during the scan as per our institutionally agreed operating procedure. Lead and device parameters were re-checked when MRI safe mode was exited. All scans were acquired in end-expiration breath-hold using retrospective ECG-gating. Following acquisition of standard pilot images, horizontal long axis (HLA), vertical long axis (VLA), and short axis (SA) cine scans were obtained. Scans were analysed off-line, using CVI 42^TM^ version 5.10.1 (Circle Cardiovascular Imaging Inc, Calgary, Alberta, Canada). Manual contouring of the LV and RV at end-systole and end-diastole was performed.

### Statistics

Statistical analysis was performed using R (version 4.3.1). All data were subjected to Kolmogorov–Smirnov tests to establish normal distribution and are presented as mean ± SD or median [interquartile range] as appropriate. Paired Student’s *t* tests were performed for paired normally distributed data and Wilcoxon signed-rank tests were performed for paired non-parametric data. Correlations were assessed using Pearson correlation analysis for normally distributed data and Spearman correlation for non-parametric data. All metabolomic and lipidomic analysis was subject to false discovery rate (set at .05) analysis using the Benjamini–Hochberg correction. A probability of *P* < .05 was considered significant (2-tailed).

## Results

### Anthropometric and baseline study data

Patients with severe NICM who were in sinus rhythm with LBBB undergoing CRT implantation were included in this study as summarized in *Table 1*. All patients were on maximal tolerated doses of goal directed HF therapy at the time of recruitment^24^ optimized over 250 [211–318] days and were median NHYA class 2.

**Table 1:** Patient demographics and prognostic heart failure medications

**Recruited (*n*)**	**14**
Age (years)	65 [61–71]
Duration of heart failure diagnosis (days)	250 [211–388]
Male	7 [50]
Weight (kg)	85 [74–99]
Body mass index (kg/m^2^)	29 [25–33]
Haemoglobin (g/L)	145 [129–150]
Estimated glomerular filtration rate (mL/min/1.73 m^2^)	77 [69–85]
Brain natriuretic peptide (pg/mL)	345 [215–386]
N-terminal pro-brain natriuretic peptide (ng/L)	2233 [1273–3404]
Neuropeptide Y (pg/mL)	24 [10–34]
Systolic blood pressure (mmHg)	122 [111–128]
Diastolic blood pressure (mmHg)	76 [67–82]
Diabetes	3 [21]
Hypertension	8 [57]
Sinus rhythm	14 [100]
Left bundle branch block	14 [100]
Baseline QRS duration (ms)	166 [162–180]
Baseline left ventricular ejection fraction (%)	30 [26–32]
Baseline New York Heart Association Class	2 [2–3]
**Heart failure medication**	
Angiotensin converting enzyme inhibitor or angiotensin receptor blocker	14 [100]
Neprilysin inhibitor	9 [64]
Mineralocorticoid receptor blocker	11 [79]
Sodium-glucose co-transporter-2 inhibitor	3 [21]
Beta blocker	12 [86]

Values are median [interquartile range] or *n* [%].

### Cardiac substrate manipulation

Altering substrate infusion resulted in significant differences in LMS concentrations and cardiac uptake (see Supplementary data online, *Figures S1* and *S2*). I + G infusion resulted in hyperinsulinaemia compared with intralipid (151.9 [87.9–259.3] vs. 54.3 [30.3–67.0] pmol/L, *P* < .01) and a small but significant increase in glucose concentration (5.76 [5.26–6.86] vs. 5.40 [4.91–6.18] mmol/L, *P* = .02) whilst intralipid significantly increased NEFA concentration (3.39 [2.60–4.24] vs. 0.78 [0.43–1.26] mmol/L, *P* < .001), and circulating ketones (BOHB rising from 0 [0–0] to 0.11 [0–0.18] mmol/L, *P* = .004). During baseline intrinsic conduction, cardiac glucose uptake was numerically but not statistically higher on I + G and intralipid infusions (59.8 [44.5–112.0] vs. 39.5 [19.5–75.3] mmol/min, *P* = .11), whilst intralipid caused significantly higher cardiac NEFA uptake (52.4 [30.0 −106.3] vs. 1.8 [0–13.0] mmol/min, *P* = .01). During intrinsic conduction, no significant differences in cardiac uptake of BOHB or lactate were observed irrespective of substrate supply (see Supplementary data online, *Figure S3*).

### Acute cardiac haemodynamic and oxygen consumption changes in response to CRT

CRT acutely shortened QRSd from 166 [162–180] to 129 [128–144] ms (*P* < .01) and changed the shape of PV loops (as shown in *Figure 2A*) leading to significant improvements in all invasive metrics of cardiac performance (stoke work, cardiac work and d*P*/d*t*_max_), irrespective of substrate supply, as summarized in *Table 2*. Cardiac contractility improved without changes in preload (LVEDP or LVEDV). CRT also resulted in significant changes to left main stem (LMS) flow, dependent on which substrate infusion was used. During intrinsic conduction, MVO_2_ was significantly higher and the respiratory quotient (RQ) significantly lower on intralipid compared with I + G infusion, with a trend towards reduced cardiac oxygen efficiency (see Supplementary data online, *Figure S3*) as would be expected with FA oxidation.^25^ However, CRT did not significantly change MVO_2_ on either infusion, leading to a significant increase in cardiac oxygen efficiency.

**Table 2:** Cardiac performance during insulin/glucose infusion and intralipid infusion during intrinsic atrioventricular node conduction and during cardiac resynchronization therapy

		Glucose			Intralipid		
	*n*	intrinsic rhythm Median [Interquartile range]	Cardiac resynchronization therapy Median [Interquartile range]	*P*	intrinsic rhythm Median [Interquartile range]	Cardiac resynchronization therapy Median [Interquartile range]	*P*
**Cardiac function**							
Stroke work (L.mmHg)	7	10.3 [7.0–11.1]	11.0 [8.6–15.1]	.**05**	8.2 [5.9–9.0]	10.8 [8.5–14.3]	.**03**
Cardiac work (L.mmHg/min)	7	552 [486–731]	715 [559–1002]	.**02**	507 [469–594]	638 [575–988]	.**02**
d*P*/d*t*_max_ (mmHg/s)	10	639 [568–707]	735 [651–798]	**<**.**01**	698 [616–718]	804 [714–867]	**<**.**01**
Left ventricular end diastolic pressure (mmHg)	10	14.3 [10.0–16.8]	11.9 [9.6–14.2]	.48	11.4 [9.2–17.5]	13.7 [11.3–18.0]	.32
Left ventricular end diastolic volume (mL)	10	286 [240–360]	277 [249–336]	.12	269 [191–321]	239 [166–309]	.89
Left main stem flow (mL/s)	11	2.9 [2.2–3.4]	2.6 [1.8–3.1]	.**05**	3.0 [2.7–3.1]	3.3 [2.6–3.5]	.**03**
**Cardiac gas exchange**							
Oxygen extraction (mL/dL)	11	6.6 [5.0–8.8]	7.5 [5.4–10.6]	.10	8.2 [6.6–13.1]	9.3 [5.5–12.7]	.49
Minute ventilation of oxygen (mL/min)	11	11.0 [6.6–14.9]	9.9 [6.3–15.9]	.32	16.6 [12.29–21.85]	16.6 [11.5–22.0]	.62
Respiratory quotient	11	1.02 [0.74–1.2]	0.96 [0.8–1.23]	1	0.91 [0.66–1.01]	0.85 [0.62–1.23]	1
**Cardiac efficiency**							
Cardiac efficiency (L.mmHg/mL)	7	65.5 [30.1–75.9]	70.7 [36.6–122.6]	.**02**	32.8 [23.1–39.3]	43 [33.5–59.2]	.**02**

*P* values are obtained from a paired Wilcoxon signed rank test and those less .05 are in bold. d*P*/d*t*_max_, peak change in left ventricular developed pressure/change in time.

### Cardiac substrate uptake in response to CRT

**Figure 3:**
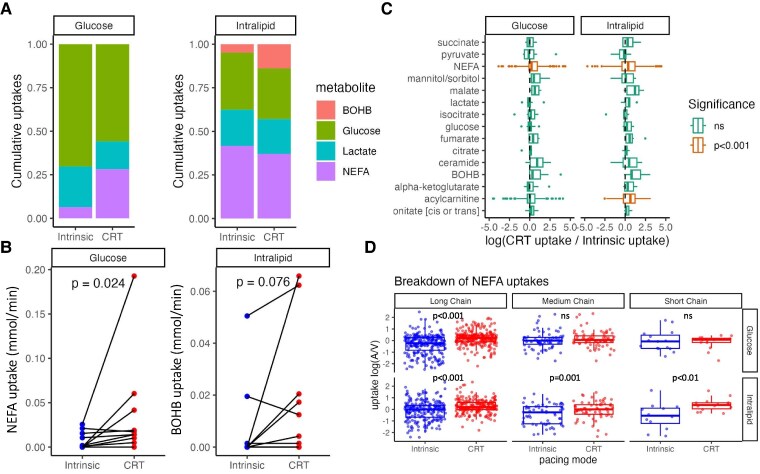
The effect of cardiac resynchronization therapy on cardiac substrate uptake during insulin/glucose and intralipid infusions. (*A*) Cumulative proportion of uptake (defined as arterio-venous concentration difference multiplied by left main coronary artery flow) of non-esterified free fatty acids, β-hydroxybutyrate, lactate and glucose during intrinsic conduction and optimized cardiac resynchronization therapy while on intralipid and insulin/glucose infusions. (*B*) Illustration of the effect of cardiac resynchronization therapy on the uptake of non-esterified fatty acid and β-hydroxybutyrate while on insulin/glucose and intralipid infusions respectively. (*C*) metabolomic analysis illustrating the difference in uptake of intermediary metabolites induced by initiating cardiac resynchronization therapy, where positive values indicate increased uptake with cardiac resynchronization therapy. (*D*) lipidomic analysis illustrating the effect of cardiac resynchronization therapy on the uptakes [defined as log (arterial/venous)] of different chain length non-esterified fatty acid while on insulin/glucose and intralipid infusions. Data from 11 patients with *P* values obtained from paired Wilcoxon signed rank tests (*B, C,* and *D*) and adjusted for false discovery rate with Benjamini–Hochberg correction (*C* and *D*)

*Figure 3A* and Supplementary data online, *Figure S4* illustrate the changes in substrate uptake following 2 min of biventricular pacing. Whilst on I + G infusion, CRT caused a significant increase in NEFA uptake from 1.8 [0–13.0] to 14.0 [7.6 −30.2] mmol/min (*P* = .02), as seen in *Figure 3B*, with no significant change in glucose, BOHB or lactate uptakes. Initiation of CRT pacing whilst on intralipid infusion did not further increase overall NEFA uptake (52.4 [30.0 −106.3] to 57.6 [21.0–73.6] mmol/min with CRT, *P* = .7) suggesting that NEFA uptake was already maximized. However, there was a strong trend towards increased BOHB uptake from 0 [0–1.1] to 8.3 [0.4–19.7] mmol/min with CRT (*P* = .076), as shown in *Figure 3B*. CRT did not change glucose uptake, and importantly there was unchanged net uptake of lactate, suggesting that oxygen supply remained adequate to match MVO_2_.

To better understand the changes in metabolite uptake in response to CRT, metabolomic and lipidomic analysis of paired arterio-venous samples were analysed. This confirmed that CRT induced a significant increase in NEFA uptake on I + G (*Figure 3C*), with the increased sensitivity and power of lipidomic analysis unmasking a small but significant increase in total NEFA uptake in response to CRT whilst on intralipid. Further subclassification of NEFA by chain lengths showed that CRT increased uptake of long-, medium-, and short-chain NEFA during intralipid infusion. On I + G infusion, however, the increase in NEFA uptake was driven solely by increases in long chain NEFA uptake (*Figure 3D*), including long chain monounsaturated, polyunsaturated and saturated NEFA (see Supplementary data online, *Figure S5*). Further subclassification of NEFA uptake on both infusions is shown in Supplementary data online, *Figure S5*. On I + G infusion CRT did not result in significant changes in intermediary metabolites of glucose metabolism. However, on the intralipid infusion CRT induced a net increase in acyl-carnitine uptake, suggesting NEFA transport into the mitochondria for oxidation. Overall metabolomics analysis did not suggest increased production of toxic intermediary metabolites with CRT on either infusion.

### Correlations between acute changes in cardiac performance and substrate uptake

**Figure 4:**
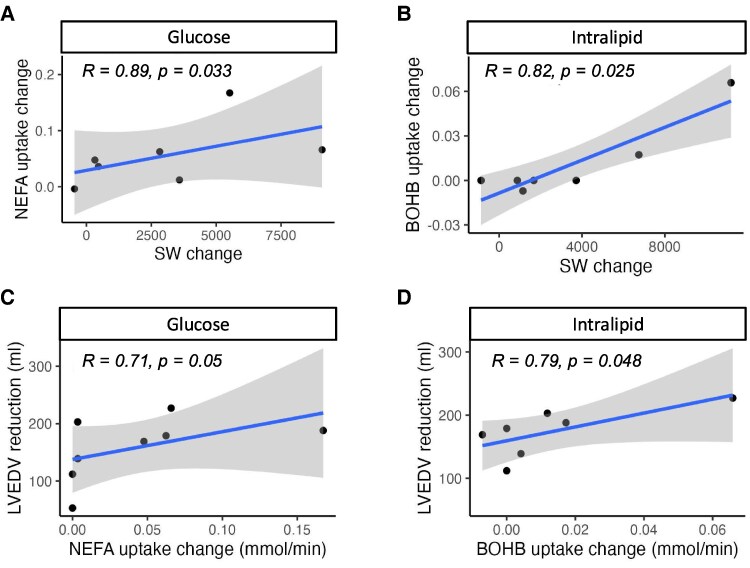
Correlations between cardiac substrate uptake during insulin/glucose and intralipid infusions and acute improvement in stroke work and subsequent reverse remodelling. The increase in stroke work in response to cardiac resynchronization therapy was significantly positively correlated with subsequent increase in non-esterified fatty acid intake during insulin/glucose infusion (*A*, *n* = 7 patients) and the increase in β-hydroxybutyrate uptake during intralipid infusion (*B*, *n* = 7 patients) after 2 min of biventricular pacing. The increase in non-esterified fatty acid (*C*, *n* = 8 patients) and β-hydroxybutyrate uptake (*D*, *n* = 7 patients) on these infusions also positively correlated with the degree of reverse remodelling as measured by the reduction in in left ventricular end diastolic volume at 6 months. *P* value obtained using Spearman correlation. Grey shaded area represents 95% confidence interval limits

A strong correlation was observed between the acute improvement in cardiac haemodynamic performance induced by QRSd shortening from CRT, and the subsequent changes in substrate uptake as summarized in Supplementary data online, *Table S1*. Whilst on I + G infusion, there was a strong positive correlation between the increase stroke work (SW) and NEFA uptake, whilst on intralipid infusion, increase in SW, cardiac work and d*P*/d*t*_max_ positively correlated with BOHB uptake as shown in *Figure 4A* and *B*. There was no significant correlation between measures of cardiac performance and other substrate uptakes (as shown in Supplementary data online, *Table S1*).

### Chronic left ventricular reverse remodelling in response to CRT

QRSd, LV volumes, LVEF, EQ-5D-3L, and NYHA class were assessed at baseline and at 219 [209–224] days follow-up. CRT resulted in significant shortening of QRSd (166 [162–180] to 136 [124–144] ms, *P* < .01), reverse LV remodelling with significant reduction in LVEDV (268 [244–322] to 142 [102–155] mL, *P* < .01) and improvement in LVEF (30 [26–33] to 40 [34–54]%), and improvement in symptoms and functional status with improved NYHA class (2 [2–3] to 2 [2–2], *P* = .04) and EQ-5D-3L score (73 [64–76] to 80 [78–91], *P* = .02).

**Figure 5:**
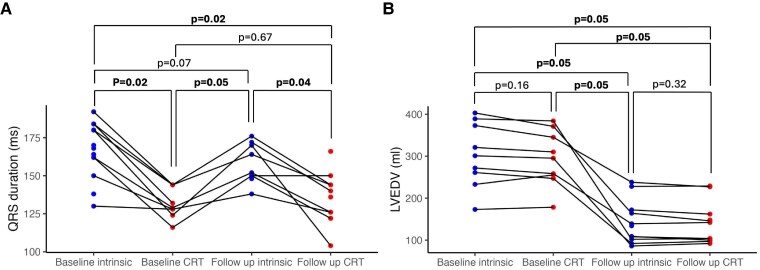
Chronic left ventricular remodelling after 6 months cardiac resynchronization therapy, independent of acute changes in QRS duration. QRS duration (*A*, 13 patients) and left ventricular end diastolic volume (*B*, 9 patients) at baseline implant and 6 month follow up, with intrinsic rhythm and optimized cardiac resynchronization therapy pacing. Baseline left ventricular end diastolic volume measurements were obtained from invasive pressure–volume loop catheters at the time of cardiac resynchronization therapy implant and follow-up measurements were obtained from cardiac MRI. *P* values were obtained from paired Wilcoxon signed rank tests and are adjusted for false discovery rate with Benjamini–Hochberg correction

QRSd was assessed at baseline (baseline intrinsic), immediately following CRT implantation with optimized pacing (baseline CRT) and at 6 month follow up with both intrinsic rhythm (follow-up intrinsic) and optimized CRT pacing (follow-up CRT). CRT pacing resulted in a significant acute reduction in QRSd, which was similar at baseline and follow up, with no differences in intrinsic QRSd between baseline and follow up as shown in *Figure 5A*. However, LVEDV at the same time points, derived from invasive PV loop volumes at implant and CMR at follow up, showed no acute reduction in LVEDV at CRT implant. However, there was a significant and substantial (48% ± 19%, *P* < .001) reduction in LVEDV at follow up, which persisted despite acutely turning CRT off resulting in lengthening of the QRSd as shown in *Figure 5B*.

### The relationship between CRT induced substrate uptake and subsequent reverse remodelling

The change in substrate uptake in response to CRT correlated with long term reverse remodelling of LVEDV as summarized in *Figure 4C* and *D* and Supplementary data online, *Table S2*. There was a significant correlation between the increase in NEFA uptake on I + G and reduction in LVEDV at follow up (*Figure 4A*). Lipidomic analysis showed this was driven by increases in both long chain (saturated, monounsaturated, and polyunsaturated) and medium chain FAs (see Supplementary data online, *Table S3*). On intralipid infusion, there was also a significant correlation between the increase in BOHB uptake and reduction in LVEDV at follow up (*Figure 4D*). There was no significant correlation between the degree of acute QRSd shortening and chronic LVEDV remodelling (see Supplementary data online, *Figure S6*), although both the acute improvement in stroke and cardiac work did correlate with LVEDV remodelling (see Supplementary data online, *Figure S7*).

## Discussion

The main findings of this study are that, firstly, in severe NICM the heart retains significant metabolic flexibility and can adapt substrate uptake in response to changes in substrate supply and CRT. Secondly, CRT results in both acute and long-term improvements in cardiac function. Acutely CRT through shortening of QRSd and reducing mechanical dys-synchrony increases SW and cardiac work and improves oxygen efficiency regardless of substrate manipulation, and in the long-term results in a dramatic reduction in LVEDV beyond that achievable through acutely varying QRSd. Thirdly, after only 2 min of biventricular pacing CRT induces changes in cardiac substrate uptake, the degree of which correlates with chronic reverse remodelling of LVEDV. Overall, in NICM, CRT reverses the metabolic phenotype of HF towards a more physiological picture of NEFA-predominant uptake. This metabolic shift is energetically favourable and may ultimately enable reverse remodelling by restoring myocardial ATP supply.

### The acute effects of CRT on cardiac metabolism

To our knowledge, this is the first study to invasively measure absolute cardiac metabolic substrate uptake through paired arteriovenous blood sampling in response to CRT. During I + G infusion cardiac glucose uptake was unchanged by CRT. However, CRT resulted in a significant increase in NEFA uptake, driven by increases in long chain monounsaturated, polyunsaturated and saturated NEFA uptake, after only 2 min of biventricular pacing. Furthermore, the magnitude of increase in NEFA uptake correlated with the acute contractile improvement in cardiac function. Importantly, NEFA uptake increased without changes in arterial NEFA supply, suggesting an active element to cardiac substrate selection induced by CRT. Overall CRT seems to reverse the glucose metabolism pre-dominant phenotype associated with HF towards a more NEFA predominant phenotype seen in healthy hearts.

During intralipid infusion, there was significantly increased cardiac NEFA uptake during baseline intrinsic conduction, and lipidomic analysis unmasked a small but significant increase in the uptake of long-, medium-, and short-chain NEFA in response to CRT (see Supplementary data online, *Figure S5*). Of note, during these conditions of near maximized NEFA uptake, the heart still demonstrated residual metabolic flexibility with a strong trend towards increased BOHB uptake induced by CRT. Furthermore, the observed increase in BOHB uptake correlated with improvements in chronic LV remodelling. This supports the notion that the increased use of ketones as an additional source of ATP in the failing heart is an active compensatory mechanism and can contribute to reverse remodelling.^26–28^ This is particularly noteworthy, given increasing interest in the therapeutic potential of ketones in HF, which have been shown to improve LVEF and cardiac output without effecting cardiac efficiency.^29^ Whilst the metabolic effects of ketones on the myocardium are likely beneficial in NICM, it is likely that additional factors contribute to the improvements in cardiac function and remodelling. For example, chronic oral ketone ingestion has been shown to improve cardiac output and LVEF, but at the same time cause a reduced pulmonary capillary wedge pressure suggesting an additional vasodilatory effect.^30^

While the beneficial effect of CRT on cardiac efficiency has been previously demonstrated,^31^ to our knowledge this is the first study to invasively measure cardiac efficiency in response to acutely altering substrate supply and initiating CRT. This is important when considering cardiac substrate selection, due to inherent differences in cardiac energy efficiency depending on substrate. Glucose is the most oxygen efficient substrate (work/MVO_2_)^32,33^ producing more ATP per mole of O_2_ consumption than either FA or ketone oxidation.^34,35^ As would then be expected, during intrinsic conduction a significantly higher MVO_2_ was observed and a lower RQ during intralipid compared with I + G infusion, associated with a trend towards reduced cardiac efficiency, validating previous findings in animal models.^36,37^ However, as oxygen delivery is not limited in NICM, CRT acutely improved cardiac efficiency irrespective of substrate infusion.

Furthermore, the lack of change in MVO_2_ and RQ with CRT despite significant subsequent changes in substrate uptake is a novel finding. The improvement in acute haemodynamic performance and efficiency may be primarily driven by the shortening of QRSd and reduction in mechanical dys-synchrony directly, rather than being secondary to the change in substrate utilization alone. On I + G infusion the increase in NEFA uptake if coupled to β-oxidation and mitochondrial ATP production would be predicted to increase MVO_2_ and reduce RQ. However, while the increase is NEFA uptake is large in absolute terms, NEFA uptake remains modest in relative terms compared with glucose uptake (*Figure 4A*) and may therefore not be sufficient to result in a measurable change in MVO_2_ or RQ. This observation is important as it suggests that even relatively modest increases in NEFA utilization may lead to improved cardiac function but without significantly reducing cardiac efficiency. Finally, during CRT on both substrate infusions, lactate uptake rather than production was seen suggesting that the heart was never oxygen limited irrespective of MVO_2_. Thus, in NICM, the traditional metric of fuel efficiency (ATP/O_2_) might be less relevant, compared with the ATP efficiency (ATP/2 carbon moiety) of the substrate being metabolized. Indeed NEFA oxidation produces over 50% more ATP per 2-carbon moiety than glucose oxidation.^25,35^ This substantial increase in ATP availability for contractile function might explain the improved cardiac contractility and energetics associated with increased NEFA uptake^10^ and ultimately enable reverse remodelling.

### Reverse remodelling

This is the first study to accurately track reverse remodelling from implant using CMR calibrated PV loop measurements and paired CMR data 6 months later during both intrinsic conduction and biventricular pacing to tease out the relationship between QRSd shortening and chronic reverse remodelling. The recent availability of MRI conditional CRT devices that also allow for programmable pacing during CMR now allows this novel approach to be taken.

An absolute reduction in LVEDV and improvement in LVEF were used as measures of reverse remodelling, in line with previous CRT trials.^12,13,38^ The 48% ± 19% reduction in LVEDV observed in the present study compares with a 10%–20% reduction seen in MADIT-CRT^17^ and MIRACLE.^38^ The 43 [29–72]% relative (10 [5–24]% absolute) improvement in LVEF, was also greater than MIRACLE (15% relative) but similar to MADIT-CRT (45% relative and 11% absolute). It is worth noting that in the present study, all patients had NICM and broad LBBB and are therefore more likely to respond to CRT,^15^ and the evolution of CRT programming and quadripolar LV lead technology since MIRICLE and MADIT-CRT has also improved CRT response rates.^15^ Furthermore, historical trials used echocardiography to assess reverse remodelling which is known to underestimate cardiac volumes and function compared with CMR, which is now recognized as the gold standard.^39^ Biventricular pacing during CMR scanning, has also been shown to result in a higher measured LVEF and smaller LV volumes as opposed to intrinsic conduction (with AOO pacing).^40^

During implant, acute shortening of the QRSd did not lead to acute changes in LVEDV. Others have reported very small reductions in LVEDV index (via transthoracic echocardiography) in the days following CRT implantation prior to discharge (−1.21 ± 12.37 mL/m^2^), although this did not correlate with subsequent reverse remodelling.^41^ At follow up, the observed chronic improvements in LVEDV were again unchanged by acutely changing QRSd by altering pacing mode (BiV vs. AOO pacing). This suggests that while CRT acutely improves electrical synchrony (as determined by QRSd) and cardiac haemodynamics, this is not sufficient to explain the degree chronic remodelling observed with CRT. Indeed, the fact that chronic remodelling persists at follow up despite acute broadening of QRSd implies it is long-term metabolic and cellular changes triggered by resynchronization that ultimately underlie reverse remodelling. However, it seems that ongoing CRT is required to sustain cellular changes, as evidenced by the gradual decline in LV function and increase in LVEDV seen when CRT is discontinued in super-responders.^42^

### Cardiac substrate uptake and reverse remodelling

The degree of acute improvement in cardiac contractility induced by biventricular pacing, shortening of QRSd and correction of dys-synchrony was correlated to chronic remodelling, as has been previously described.^43^ However, the subsequent changes in cardiac substrate uptake induced by 2 min of biventricular pacing closely correlated with longer term reverse remodelling despite not acutely changing MVO_2_. Specifically, the increase in NEFA uptake during I + G infusion and increase in BOHB uptake while on intralipid infusion correlated with chronic remodelling of LVEDV (*Table 2* and *Figure 4D* and *E*).

If the failing heart is indeed ATP starved, then it can be hypothesized that reverse remodelling ultimately relies on the heart’s ability to augment ATP supply to the cardiac contractile machinery or improve efficiency to couple ATP generation with use for contraction. In NICM, the heart retains a degree of metabolic flexibility to alter substrate use to match arterial supply and changes in workload.^10^ In the presence of preserved metabolic flexibility, CRT further alters cardiac substrate use towards a healthy phenotype of NEFA predominance. Furthermore, in the context of maximized NEFA uptake, BOHB uptake was increased during CRT. In the absence of oxygen limitation, the favourable ATP efficiency of NEFA (and BOHB) metabolism may improve ATP availability in cardiomyocytes ultimately allowing for reverse remodelling to occur. Indeed, NEFA use leads to improved myocardial energetics and contractility, and the beneficial haemodynamic effects of BOHB use have also been demonstrated previously.^29^ Furthermore, the shift towards NEFA metabolism has been shown to persist even after 6 months of CRT, where it is also associated with improved mitochondrial function.^44^ Interestingly, high baseline cardiac NEFA uptake in an NICM may be associated with poorer response to CRT.^45^ However, it has been argued that high baseline cardiac NEFA uptake may limit the ability to further increase uptake in response to CRT, thus limiting remodelling.^46^ Indeed, this was observed during intralipid infusion.

### Limitations

These findings are from a relatively small number of participants and due to the high complexity and challenging nature of the protocol, full datasets were not available for every participant. However, the strength of the study lies in its uniqueness and the detailed phenotyping of each participant, with paired data not only on different substrate infusions, but also at implant and follow up. There was a relatively low level of SGLT2i use amongst participants (21%) as they were recruited prior to their use being incorporated into international guidelines and being widely adopted, and one patient was initiated on an SGLT2i towards the end of the follow up period. However, recent data suggest that CRT induces similar degrees of reverse remodelling in patients with and without SGLT2i.^47^ Part of the beneficial impact of SGLT2i on reverse remodelling may be related to their impact on iron metabolism and sympathetic nerve activity.^48^ None of the patients were anaemic, experienced a significant drop in Hb or underwent an iron infusion during the follow up period in the present study. The sympathetic co-transmitter neuropeptide-Y has also recently been identified as a prognostic marker in HF, above and beyond BNP levels^49^ although there is no correlation with NPY levels and subsequent reverse remodelling in the present study (*R* = 0.21, *P* = .62).

Measured coronary flow and arterial blood sampling was only from the LMS and so does not consider right coronary artery blood flow and substrate/oxygen delivery. Similarly, CS venous sampling does not account for all venous drainage, with 20%–30% of venous blood draining directly into the cardiac chambers via the Thebesian veins in the lesser cardiac venous system.^50^ All participants underwent intralipid infusion after the euglycaemic clamp. This has the potential for systematic bias, but the primary objective of the infusion, that is, to deliver excess substrate to the myocardium and delineate residual flexibility, was demonstrated. It is not possible to directly measure myocardial substrate oxidation *in vivo*, changes in cardiac substrate use were inferred from cross-heart arteriovenous substrate concentration differences. However, concomitant changes in MVO_2_, RQ, and intermediary metabolites were also demonstrated.

Finally, patients also underwent CMR during atrial pacing at 10 bpm above intrinsic atrial rate to prevent competitive pacing. We feel that this is unlikely to account for the magnitude of change in LVEDV observed 6 months following CRT implantation (48% reduction), and the comparison on intrinsic AV conduction vs. CRT pacing is at the same heart rate at baseline and 6 months.

## Conclusions

Overall, in NICM the heart retains substantial metabolic flexibility, and CRT is thus able to reverse the metabolic phenotype of HF towards a more physiological picture of NEFA uptake, and even promote BOHB uptake once NEFA uptake is maximized. The degree of retained metabolic flexibility is correlated with long-term reverse remodelling of LVEDV. Ultimately cardiac remodelling in response to CRT seems to rely on changes in cellular metabolism triggered by cardiac resynchronization, rather than the acute effects of QRSd narrowing.

## Supplementary Material

ehaf033_Supplementary_Data

## Declarations

### Data Availability

The original data that support the findings of this study are available from the corresponding author upon reasonable request.
