# Supplementary material for: Metabolic flexibility and reverse remodelling of the failing human heart
Source: Eur Heart J. 2025 Feb 25;46(25):2422–33. doi: 10.1093/eurheartj/ehaf033 (PMC12208774; doi:10.1093/eurheartj/ehaf033)
Supplement: ehaf033_Supplementary_Data [file ehaf033_supplementary_data.pptx]

## Slide 1
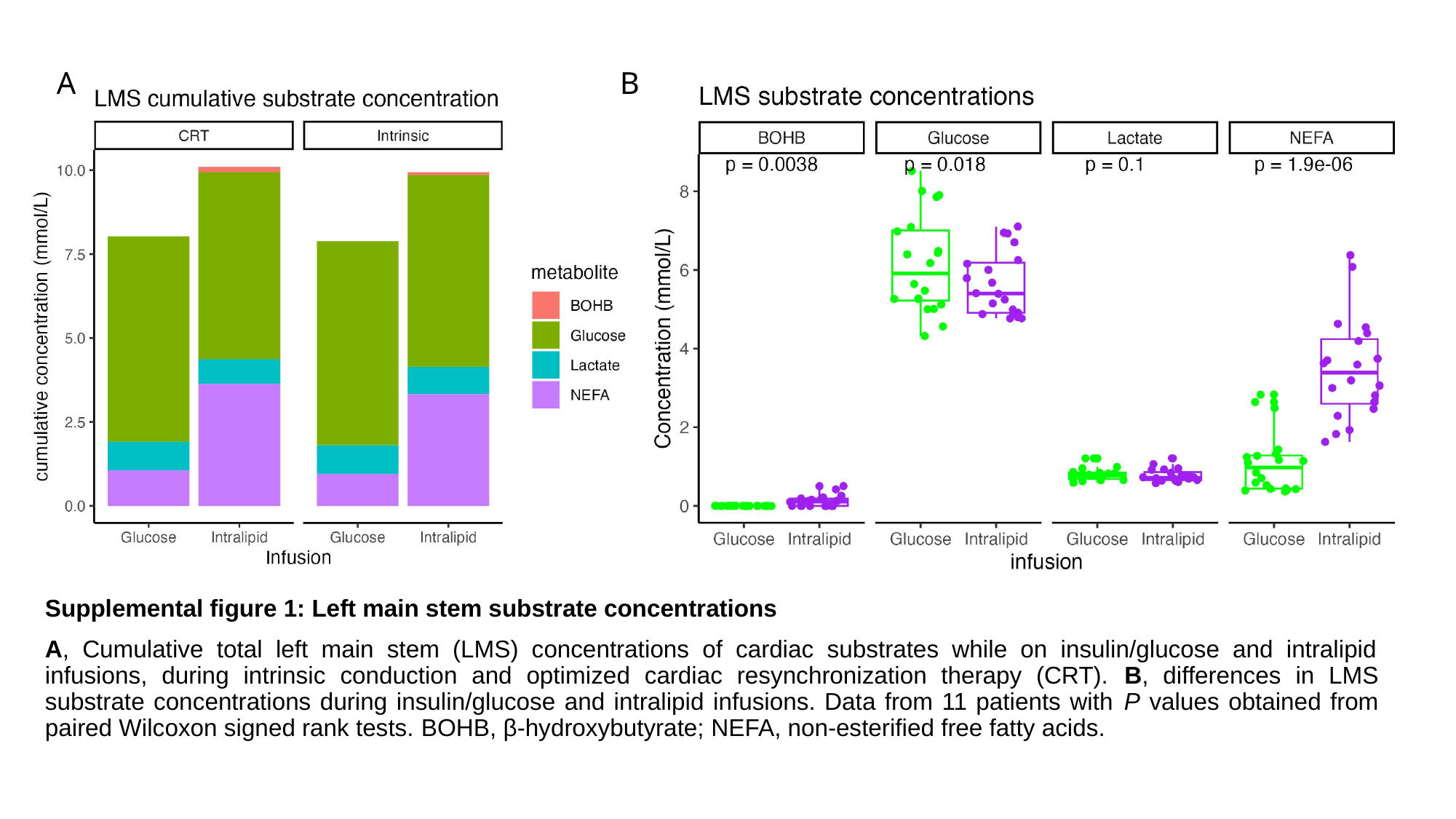

A
B
Supplemental figure 1: Left main stem substrate concentrations
A, Cumulative total left main stem (LMS) concentrations of cardiac substrates while on insulin/glucose and intralipid infusions, during intrinsic conduction and optimized cardiac resynchronization therapy (CRT). B, differences in LMS substrate concentrations during insulin/glucose and intralipid infusions. Data from 11 patients with P values obtained from paired Wilcoxon signed rank tests. BOHB, β-hydroxybutyrate; NEFA, non-esterified free fatty acids.

## Slide 2
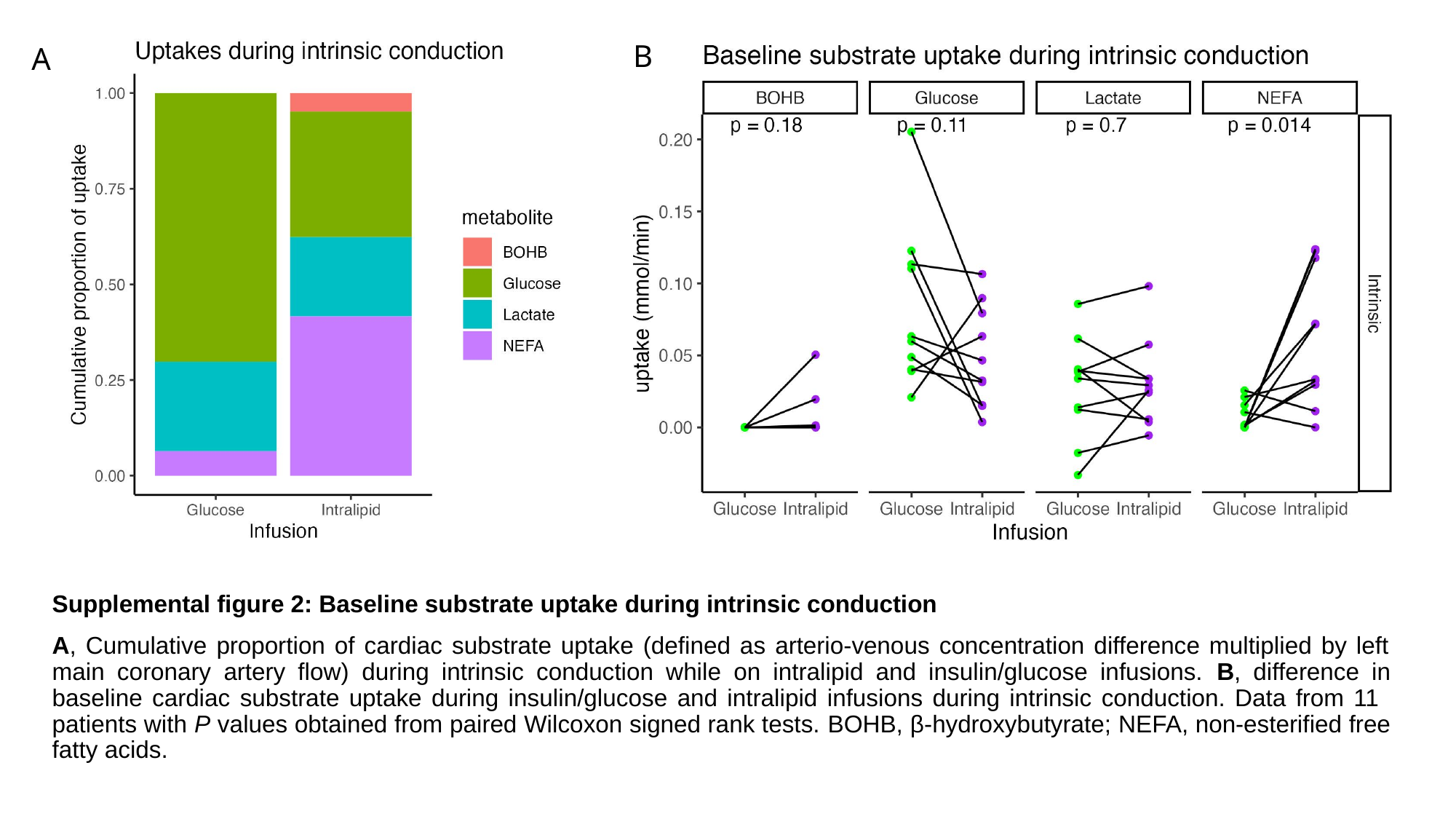

B
A
Supplemental figure 2: Baseline substrate uptake during intrinsic conduction
A, Cumulative proportion of cardiac substrate uptake (defined as arterio-venous concentration difference multiplied by left main coronary artery flow) during intrinsic conduction while on intralipid and insulin/glucose infusions. B, difference in baseline cardiac substrate uptake during insulin/glucose and intralipid infusions during intrinsic conduction. Data from 11 patients with P values obtained from paired Wilcoxon signed rank tests. BOHB, β-hydroxybutyrate; NEFA, non-esterified free fatty acids.

## Slide 3
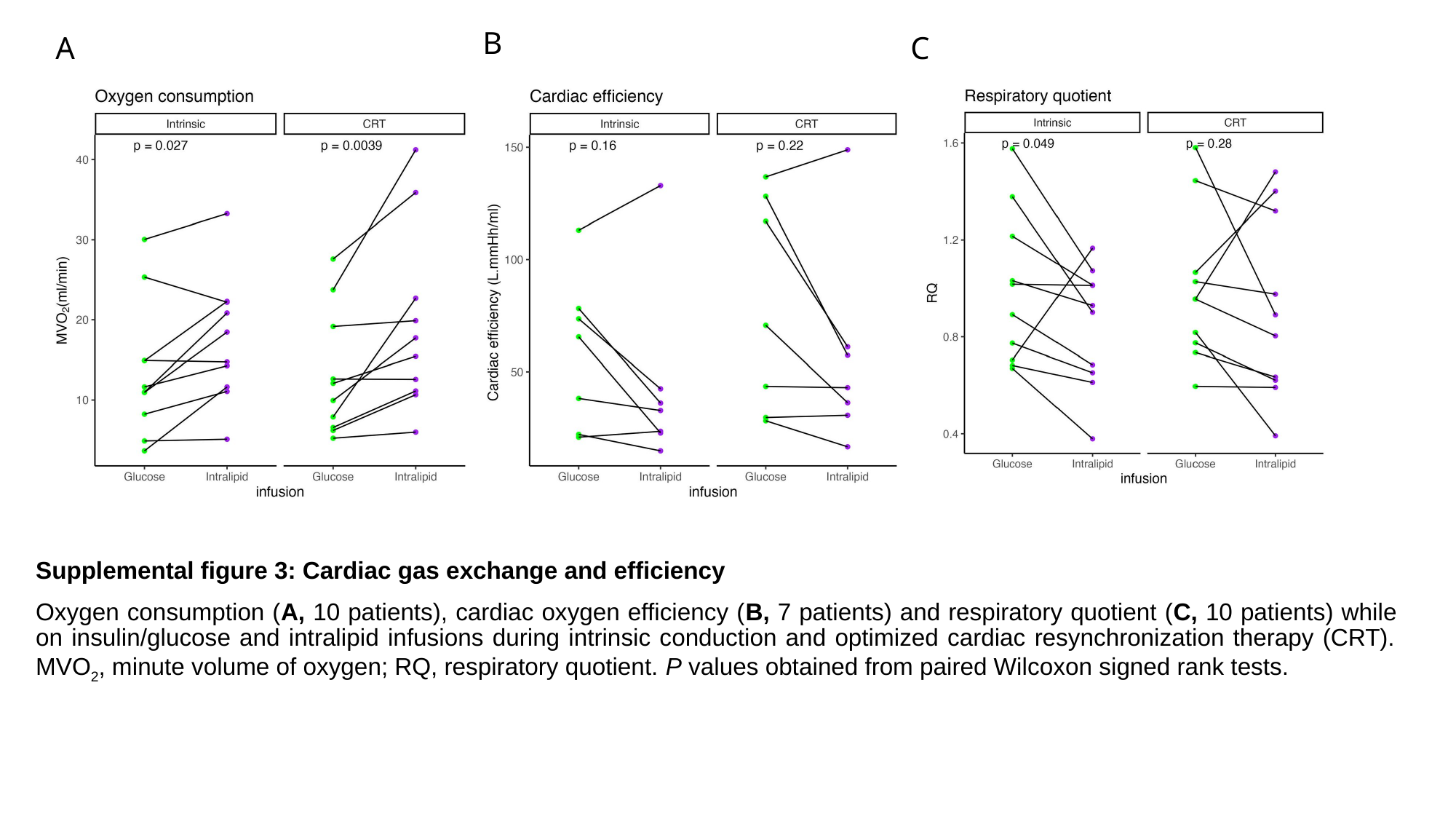

B
A
C
Supplemental figure 3: Cardiac gas exchange and efficiency
Oxygen consumption (A, 10 patients), cardiac oxygen efficiency (B, 7 patients) and respiratory quotient (C, 10 patients) while on insulin/glucose and intralipid infusions during intrinsic conduction and optimized cardiac resynchronization therapy (CRT). MVO2, minute volume of oxygen; RQ, respiratory quotient. P values obtained from paired Wilcoxon signed rank tests.

## Slide 4
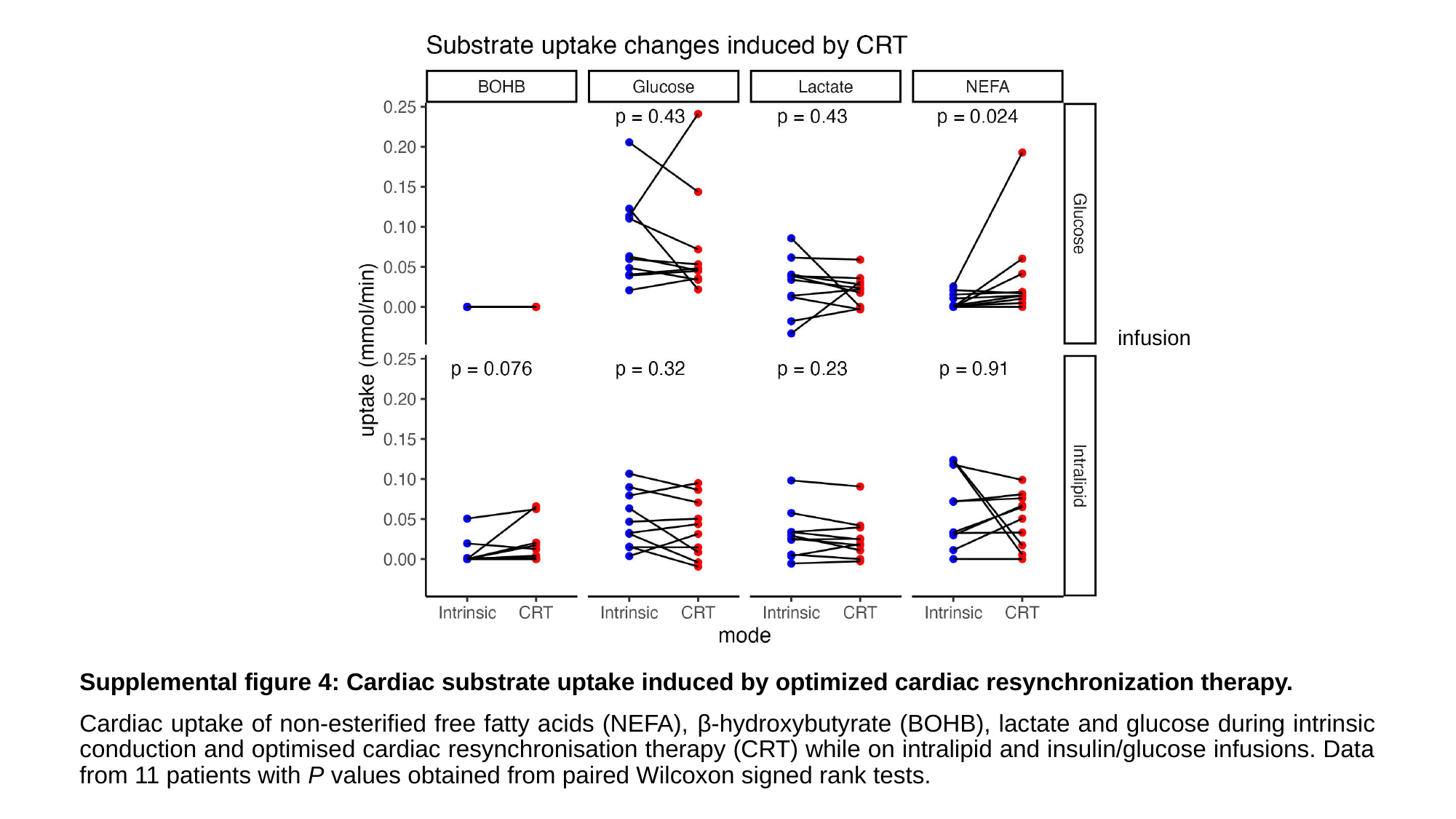

infusion
Supplemental figure 4: Cardiac substrate uptake induced by optimized cardiac resynchronization therapy.
Cardiac uptake of non-esterified free fatty acids (NEFA), β-hydroxybutyrate (BOHB), lactate and glucose during intrinsic conduction and optimised cardiac resynchronisation therapy (CRT) while on intralipid and insulin/glucose infusions. Data from 11 patients with P values obtained from paired Wilcoxon signed rank tests.

## Slide 5
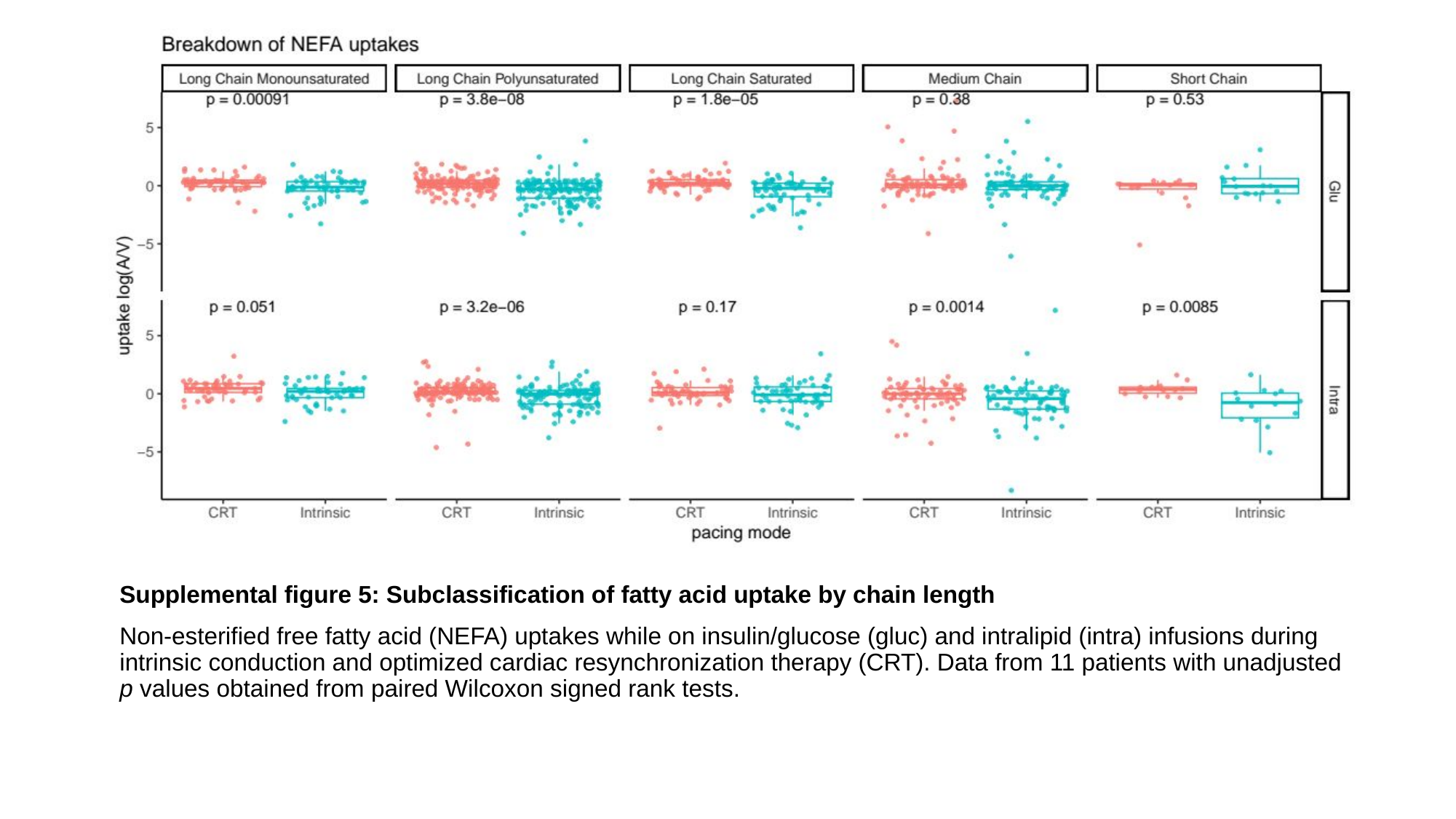

Supplemental figure 5: Subclassification of fatty acid uptake by chain length
Non-esterified free fatty acid (NEFA) uptakes while on insulin/glucose (gluc) and intralipid (intra) infusions during intrinsic conduction and optimized cardiac resynchronization therapy (CRT). Data from 11 patients with unadjusted p values obtained from paired Wilcoxon signed rank tests.

## Slide 6
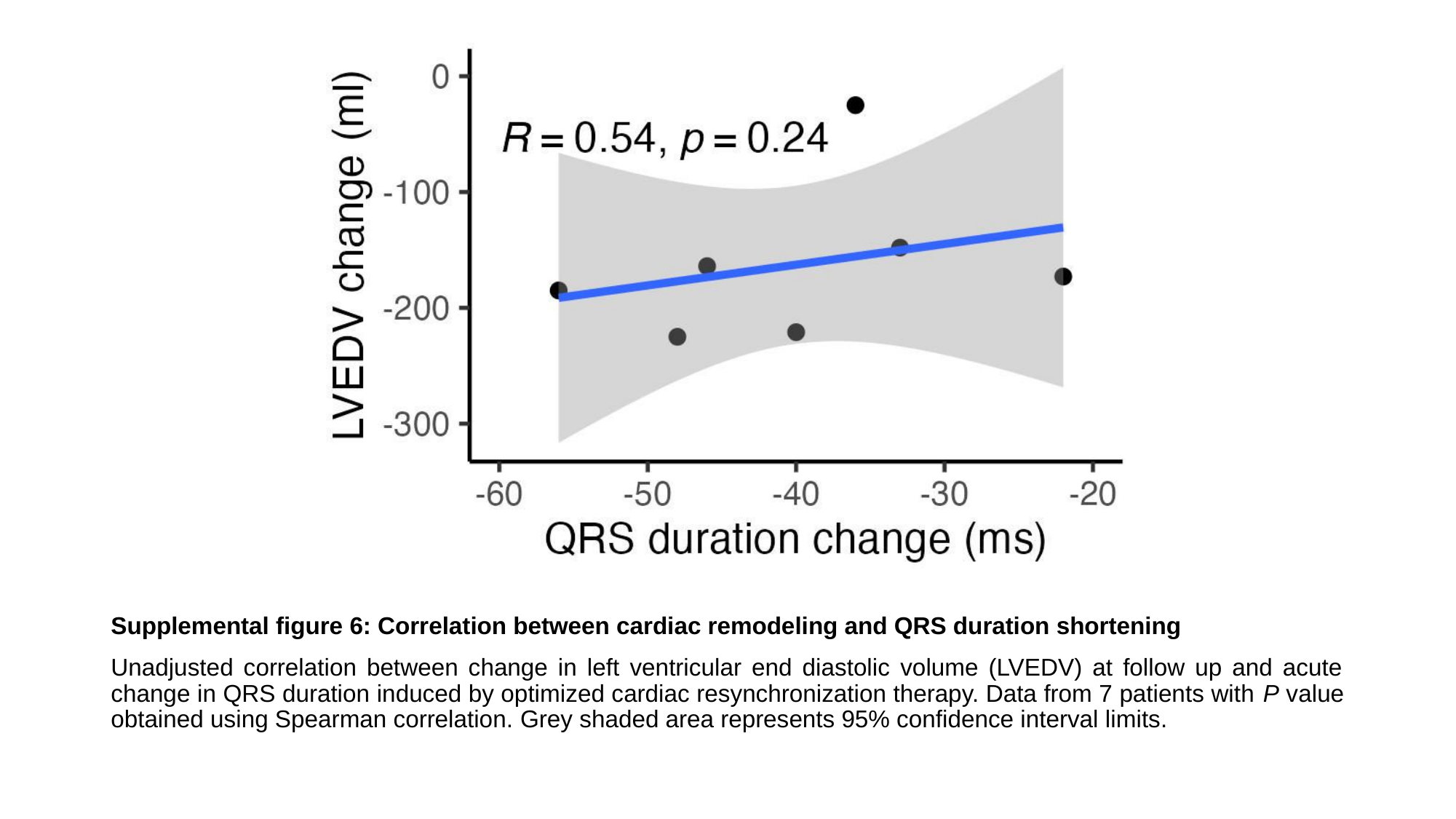

Supplemental figure 6: Correlation between cardiac remodeling and QRS duration shortening
Unadjusted correlation between change in left ventricular end diastolic volume (LVEDV) at follow up and acute change in QRS duration induced by optimized cardiac resynchronization therapy. Data from 7 patients with P value obtained using Spearman correlation. Grey shaded area represents 95% confidence interval limits.

## Slide 7
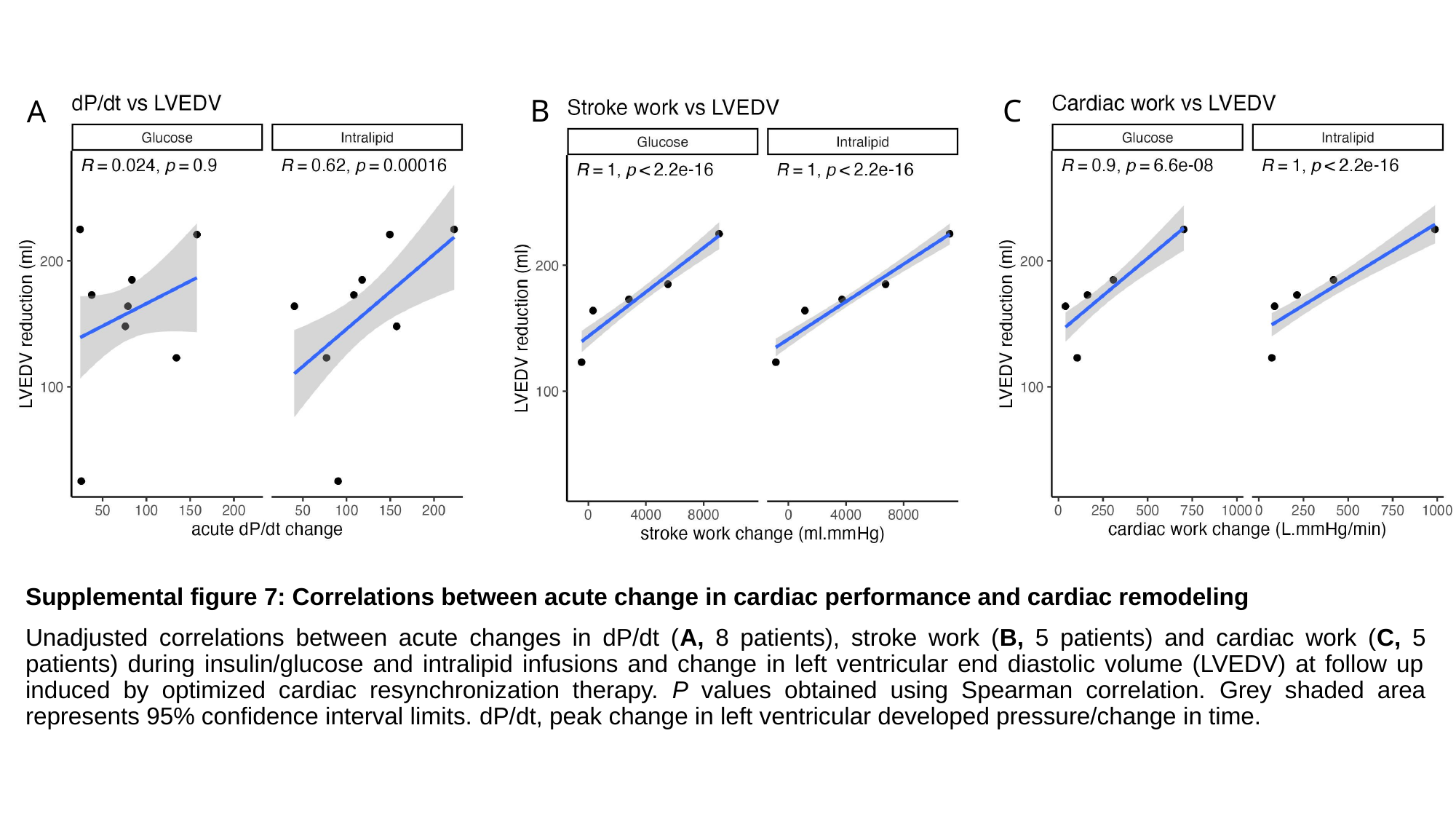

B
C
A
Supplemental figure 7: Correlations between acute change in cardiac performance and cardiac remodeling
Unadjusted correlations between acute changes in dP/dt (A, 8 patients), stroke work (B, 5 patients) and cardiac work (C, 5 patients) during insulin/glucose and intralipid infusions and change in left ventricular end diastolic volume (LVEDV) at follow up induced by optimized cardiac resynchronization therapy. P values obtained using Spearman correlation. Grey shaded area represents 95% confidence interval limits. dP/dt, peak change in left ventricular developed pressure/change in time.

## Slide 8
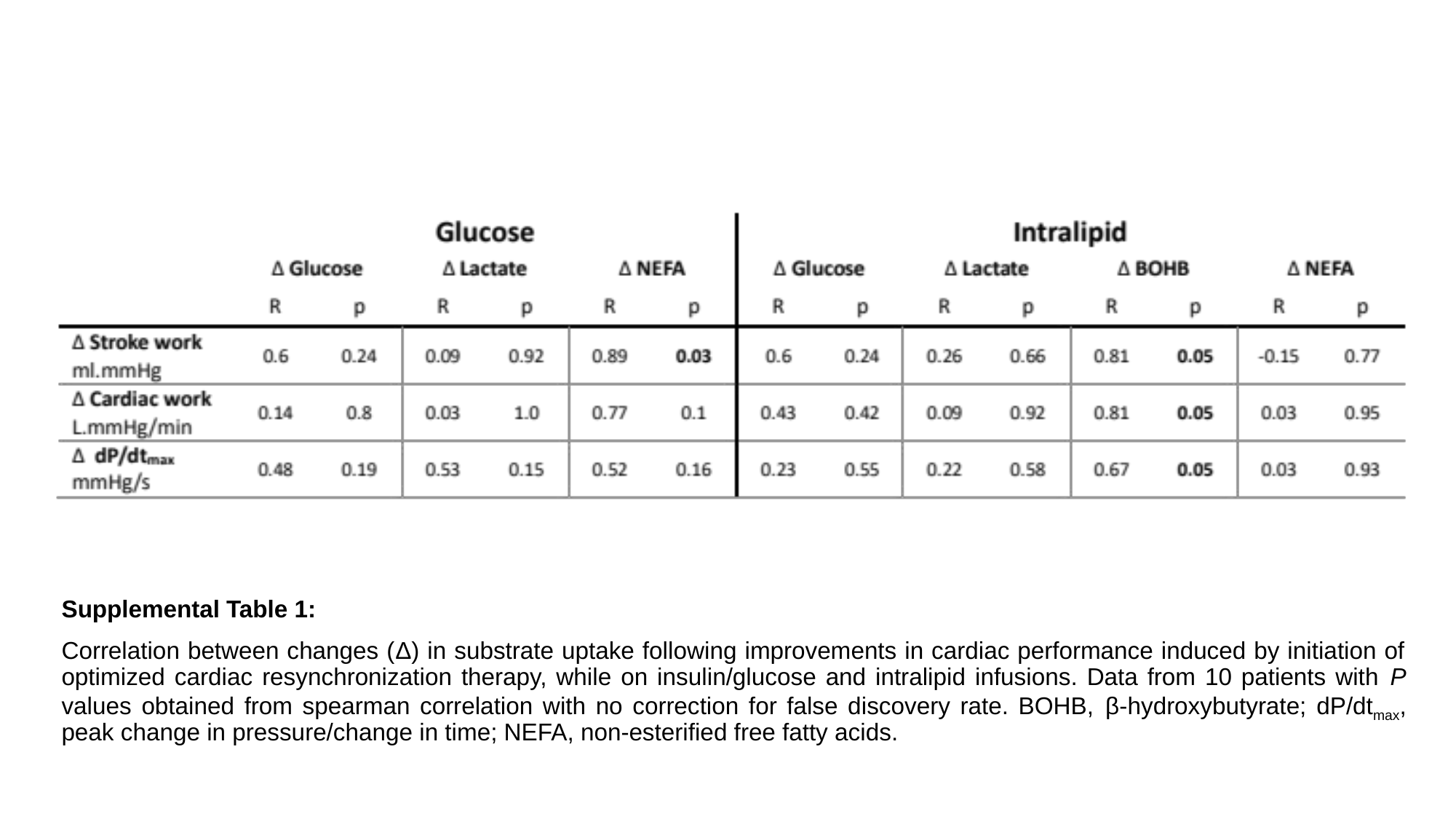

Supplemental Table 1:
Correlation between changes (Δ) in substrate uptake following improvements in cardiac performance induced by initiation of optimized cardiac resynchronization therapy, while on insulin/glucose and intralipid infusions. Data from 10 patients with P values obtained from spearman correlation with no correction for false discovery rate. BOHB, β-hydroxybutyrate; dP/dtmax, peak change in pressure/change in time; NEFA, non-esterified free fatty acids.

## Slide 9
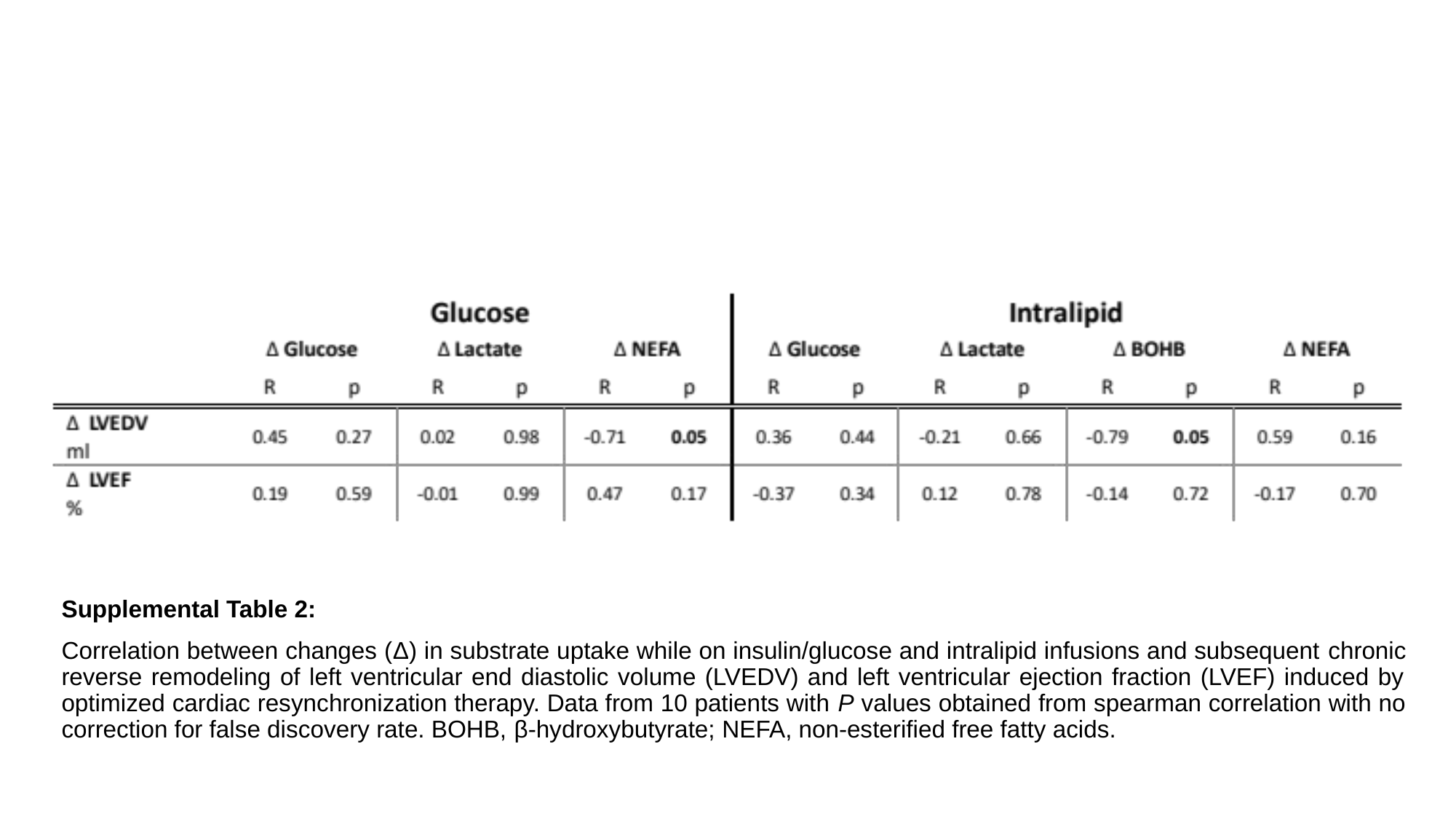

Supplemental Table 2:
Correlation between changes (Δ) in substrate uptake while on insulin/glucose and intralipid infusions and subsequent chronic reverse remodeling of left ventricular end diastolic volume (LVEDV) and left ventricular ejection fraction (LVEF) induced by optimized cardiac resynchronization therapy. Data from 10 patients with P values obtained from spearman correlation with no correction for false discovery rate. BOHB, β-hydroxybutyrate; NEFA, non-esterified free fatty acids.

## Slide 10
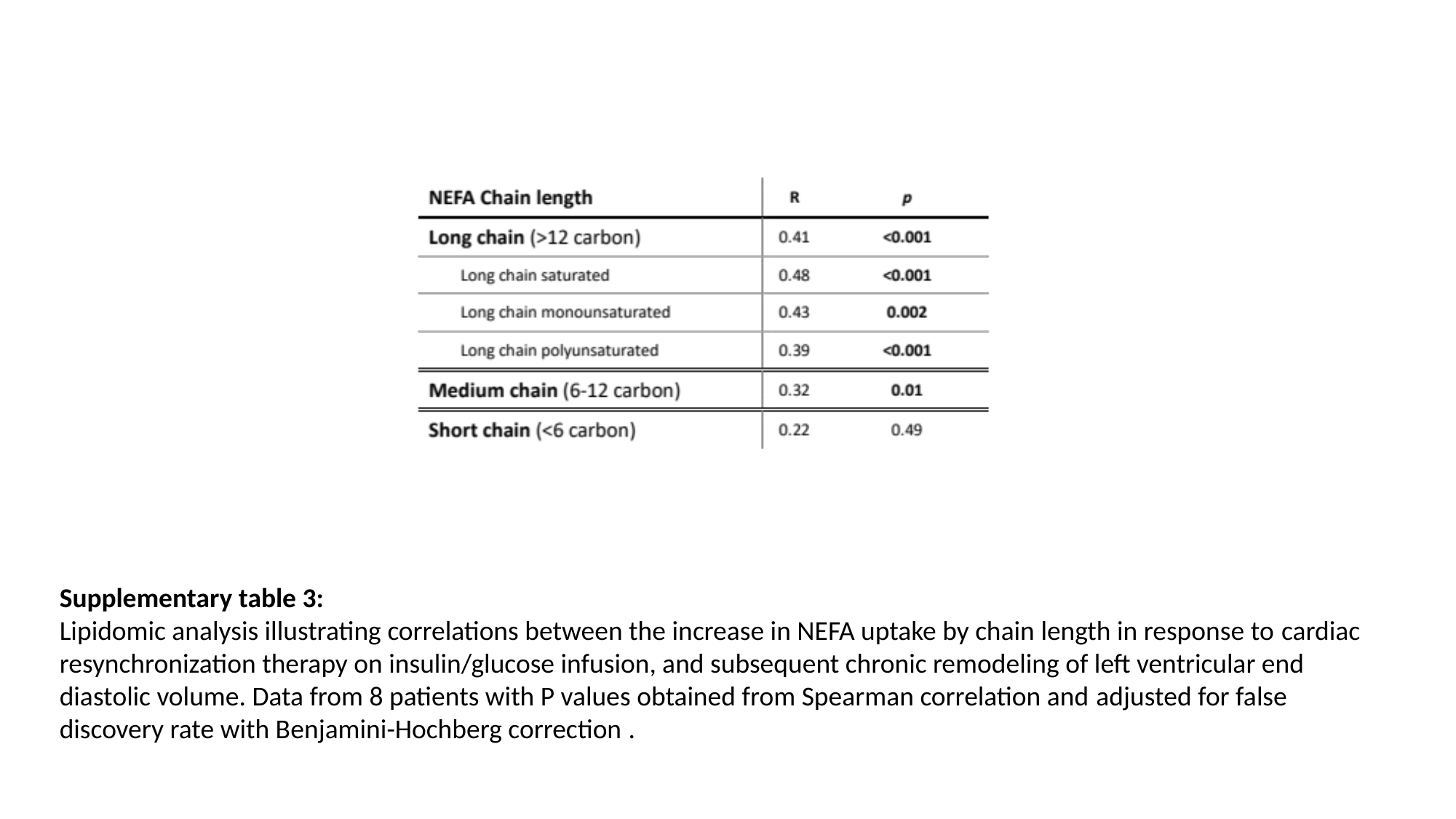

Supplementary table 3:
Lipidomic analysis illustrating correlations between the increase in NEFA uptake by chain length in response to cardiac resynchronization therapy on insulin/glucose infusion, and subsequent chronic remodeling of left ventricular end diastolic volume. Data from 8 patients with P values obtained from Spearman correlation and adjusted for false discovery rate with Benjamini-Hochberg correction .
